# Every-Other-Day Fasting Prehabilitation Attenuates Secondary Injury Following Spinal Cord Injury and Is Associated with Stage-Dependent SREBP1-Related Metabolic–Immune Remodeling

**DOI:** 10.1007/s12035-026-06139-6

**Published:** 2026-08-17

**Authors:** Yinuo Zhao, Zhongyan Xu, Nianyi Sun, Yu He

**Affiliations:** 1 School of Medicine, Tongji University, Shanghai, China; 2 Department of Rehabilitation, Shanghai Fourth People’s Hospital, School of Medicine, Tongji University, Shanghai, China; 3 Department of Rehabilitation, Shengjing Hospital of China Medical University, Shenyang, China

**Keywords:** Intermittent fasting, Prehabilitation, Spinal cord injury, SREBP1, Fatty acid remodeling, Neuroinflammation

## Abstract

Spinal cord injury (SCI) induces secondary damage characterized by metabolic disturbance, excessive inflammation, and progressive neurological dysfunction. Every-other-day fasting (EODF) has shown neuroprotective potential, but the mechanisms underlying its effects in SCI remain unclear. We investigated whether EODF prehabilitation attenuates SCI-associated secondary injury through SREBF1-associated metabolic–immune remodeling. Female C57BL/6 J mice underwent EODF for 4 weeks before SCI induction, and adeno-associated virus-mediated SREBF1 knockdown was used to assess the functional contribution of SREBF1. EODF was well tolerated, significantly improved locomotor recovery, reduced lesion cavity formation, and attenuated histopathological damage after SCI. EODF also had stage-dependent effects on SREBF1 expression, suppressing its aberrant early protein elevation while promoting coordinated regulation at later stages. In parallel, EODF partially restored altered omega-3 and omega-6 fatty acid profiles, attenuated TLR4/MyD88 signaling, reduced TNF-α, increased IL-10, shifted macrophage/microglia-associated markers toward a less proinflammatory profile, and reduced the proportion of TUNEL-positive cells. These protective effects were markedly attenuated by SREBF1 knockdown. Overall, our findings indicate that EODF prehabilitation mitigates SCI-associated secondary injury and support SREBF1-associated metabolic–immune remodeling as a functionally relevant mechanism underlying this protection. The results further suggest that temporal regulation of metabolic–inflammatory coupling may contribute to spinal cord resilience after injury.

**Supplementary Information:**

The online version contains supplementary material available at 10.1007/s12035-026-06139-6.

## MIntroduction

Spinal cord injury (SCI) is a serious neurological disorder that often results in persistent motor, sensory, and autonomic dysfunction [1]. In addition to the initial mechanical insult, secondary injury processes substantially influence neurological outcomes [2, 3]. These processes include ischemia, oxidative stress, metabolic disturbance, and uncontrolled inflammatory responses, which amplify tissue damage and impair recovery after SCI [4–6]. Therapeutic approaches have therefore targeted key components of secondary injury. For example, CHBP-loaded mesoporous polydopamine nanoparticles have been developed to attenuate neuroinflammation and modulate microglia–neuron crosstalk as a potential strategy for promoting spinal cord repair [7]. However, few interventions broadly limit secondary injury, and strategies that increase spinal cord resilience before or after injury are needed.

Prehabilitation refers to interventions applied before injury or surgery to improve tissue tolerance to an anticipated insult [8]. Among these strategies intermittent fasting (IF), particularly every-other-day fasting (EODF), has received attention for its effects on energy metabolism, inflammatory regulation, oxidative stress, and cellular adaptation [9, 10]. Fasting-related interventions have shown neuroprotective effects in several central nervous system disorders [11, 12], including SCI [13]. However, the molecular mechanisms by which EODF prehabilitation influences SCI-associated secondary injury remain incompletely understood.

Metabolic dysregulation is an important feature of SCI pathology, and altered lipid metabolism is closely linked to postinjury inflammation [14]. Sterol regulatory element-binding protein 1 (SREBP1) is a central transcriptional regulator of lipid synthesis and fatty acid homeostasis [15]. Evidence suggests that, beyond its metabolic role, SREBP1 also modulates inflammatory responses through lipid-associated signaling pathways [16]. After SCI, persistent activation of proinflammatory macrophages and microglia contributes to immune imbalance and secondary tissue damage [17, 18]. SREBP1 may therefore link metabolic disturbance to immune dysregulation after SCI. However, whether EODF prehabilitation confers protection through SREBP1-associated modulation of fatty acid balance and inflammatory signaling remains unknown.

In the present study, we tested the hypothesis that EODF prehabilitation attenuates secondary injury after SCI through SREBF1-associated metabolic and immune remodeling. We combined a mouse SCI model combined with adeno-associated virus (AAV)-mediated SREBF1 knockdown and evaluated functional recovery, histopathological injury, fatty acid profiles, inflammatory signaling, macrophage/microglia-related phenotypic changes, and apoptosis at multiple postinjury time points. Our findings provide evidence that EODF prehabilitation mitigates SCI-associated secondary damage and identify SREBF1 as a potential mediator linking metabolic regulation to immune remodeling after SCI.

## Materials and Methods

### Animals

Female C57BL/6 J mice (6–8 weeks old, 18–20 g) were purchased from SPF (Suzhou) Biotechnology Co., Ltd. (license No. SCXK [Suzhou] 2022–0006). Mice were housed under specific pathogen-free conditions (22 ± 2 °C, 50 ± 5% humidity, and a 12-h light/dark cycle) [19]. Routine microbiological health monitoring yielded negative results for all viral, bacterial, and parasitic agents included in the facility monitoring panel. Following 1 week of acclimatization, mice were used in subsequent experiments. All animal procedures were approved by the Animal Ethics Committee of Tongji University (approval No. TJBH07324101).

### Experimental Design and Grouping

A total of 230 mice were randomly assigned to five groups (*n* = 46 per group): sham, SCI, EODF + SCI, AAV-shScramble + SCI, and AAV-shSrebf1 + EODF + SCI. The sham group underwent laminectomy alone, without spinal cord compression. The SCI group underwent SCI without dietary or viral intervention. The EODF + SCI group received EODF prehabilitation before SCI. The AAV-shScramble + SCI group received the control virus before SCI. The AAV-shSrebf1 + EODF + SCI group received SREBF1 knockdown virus followed by EODF prehabilitation before SCI. The experimental schedule is presented in Figure S1. Investigators were blinded to group allocation during behavioral assessment and quantitative analysis when applicable.

### EODF Prehabilitation Protocol

Mice in the EODF + SCI and AAV-shSrebf1 + EODF + SCI groups underwent EODF starting at week 3 after AAV injection. The regimen consisted of alternating 24-h periods of fasting (with free access to water) and 24-h periods of ad libitum feeding for 4 consecutive weeks [12, 20]. Mice in the other groups were maintained under standard feeding conditions throughout the study. All mice were fed the same irradiated formulated maintenance chow for mice and rats (Shanghai Zhouyu Biotechnology Co., Ltd., Shanghai, China). The chow was intended for maintenance-stage feeding and was irradiated at 35 kilogray before use. The detailed label-declared composition of the chow is provided in Supplementary Table S1. The same chow was provided to all experimental groups; therefore, the EODF intervention differed only in feeding schedule, not in chow composition. Body weight and food intake were recorded daily at a fixed time (9:00–10:00 AM). If body weight loss exceeded 20% of baseline body weight, the fasting intervention was terminated for that animal. No such cases occurred in this study.

### SCI Model

Mice were anesthetized by intraperitoneal injection of 2% pentobarbital sodium (40 mg/kg; Xinya Biotechnology Co., Ltd., China; CAS No. 57–33-0; purity ≥ 98.0%). After dorsal hair removal and sterilization, a midline skin incision was made to expose the T10 spinal segment. After laminectomy at T10, SCI was induced using a modified forceps-compression method. Customized forceps (RWD Life Science Co., Ltd., Shenzhen, China) with a 0.35-mm tip gap were used to compress the spinal cord vertically for 30 s [21]. Successful injury was indicated by bilateral hindlimb twitching, tail spasm, and subsequent flaccid paralysis of the hindlimbs. The muscles and skin were sutured in layers after compression, and 1 mL of warm sterile saline was administered intraperitoneally to prevent shock. Postoperative care included manual bladder expression twice daily until spontaneous voiding recovered. No animal developed a suspected urinary tract infection requiring antibiotic treatment, and neither penicillin nor other antibiotics were administered during the experiment. Mice in the sham group underwent laminectomy without spinal cord compression.

### AAV-Mediated SREBF1 Knockdown

For SREBF1 knockdown, three candidate shRNA sequences targeting mouse SREBF1 were designed and cloned into a U6-shRNA vector carrying a GFP reporter cassette. The candidate shRNAs were screened in 293 T cells by cotransfecting the SREBF1 overexpression plasmid with each shRNA plasmid. Total RNA was extracted 48 h after transfection, and SREBF1 expression was quantified by RT-qPCR. Among the three candidate shRNAs, BC-1594 showed the highest knockdown efficiency and was selected for AAV9 packaging. The selected SREBF1 shRNA sequence was 5′-CAGTCTGCTTTGGAACCTCATTTCAAGAGAATGAGGTTCCAAAGCAGACTG-3′. A nontargeting scrambled shRNA sequence, 5′-CCTAAGGTTAAGTCGCCCTCG-3′, cloned into the same vector backbone was used as the negative control. Recombinant AAV9 virus carrying an shRNA targeting SREBF1 and a GFP reporter (AAV9-shSrebf1-GFP; titer, 1 × 10^12^ vg/mL) and a control virus carrying the scrambled shRNA (AAV9-shScramble-GFP) were constructed and packaged by Brain Case (Shenzhen, China) Biotechnology Co., Ltd. While anesthetized with 2% pentobarbital sodium, mice received an intrathecal injection at the L5–L6 intervertebral space. A total of 10 μL of viral suspension was injected at a rate of 1 μL per 5 s, and the needle was retained in place for 60 s before withdrawal. Mice in the AAV-shScramble + SCI group received an equivalent amount of control virus. The candidate shRNA sequences and screening results are provided in Supplementary Table S2, and validation of AAV-mediated SREBF1 knockdown in the spinal cord is presented in Figure S2.

### Sample Collection

Samples were collected at 3, 7, 14, and 21 days after SCI. For serum collection, blood was obtained by cardiac puncture under anesthesia and allowed to clot at room temperature for 30 min. The samples were then centrifuged at 3000 rpm for 15 min at 4 °C, and serum was carefully collected from the supernatant [22]. Cerebrospinal fluid (CSF, 10 μL) was collected from the cisterna magna using a glass capillary tube [23]. All serum and CSF samples were stored at − 80 °C until analysis. For spinal cord tissue collection, animals assigned to histological and immunofluorescence analyses were transcardially perfused with ice-cold phosphate-buffered saline, followed by 4% paraformaldehyde. An approximately 8-mm spinal cord segment centered on the T10 lesion epicenter, extending approximately 4 mm rostrally and 4 mm caudally, was harvested. Tissues used for histology, immunofluorescence, and TUNEL staining were fixed in 4% paraformaldehyde at 4 °C for 12 h and subsequently processed for paraffin embedding. Separate spinal cord samples used for RT-qPCR and western blotting were rapidly collected, snap-frozen in liquid nitrogen, and stored at − 80 °C until analysis [24].

### Behavior Assessment

Behavioral assessments were performed in a blinded manner at 1, 7, 14, and 21 days after SCI. Locomotor recovery was evaluated using the Basso Mouse Scale (BMS), with scores ranging from 0 to 9 [25]. Hindlimb reflex function was assessed by tail suspension, with scores ranging from 0 to 3 [26]. The inclined plane test was used to determine the maximum angle at which each mouse could maintain its position for 5 s without falling [27].

### Histological Analysis

Spinal cord tissues were fixed in 4% paraformaldehyde, dehydrated through graded ethanol, embedded in paraffin, and sectioned coronally at a thickness of 5 μm. Sections were stained with hematoxylin and eosin (HE) and examined under a light microscope [28]. Lesion cavity area was quantified using ImageJ v1.54f software (National Institutes of Health, Bethesda, MD, USA) and expressed as a percentage of the total spinal cord area. For each animal, measurements from three sections were averaged for statistical analysis. Histological analysis was performed in a blinded manner.

### Fatty Acid Measurement

Omega-3 and omega-6 fatty acid levels in serum and CSF were measured using commercial enzyme-linked immunosorbent assay (ELISA) kits. The omega-3 fatty acid kit (Catalog No. DM-X6929, Duma Biotechnology Co., Ltd., Shanghai, China) had a detection range of 0–80 μmol/L, with intra-assay variability < 8% and interassay variability < 10%. The omega-6 fatty acid kit (Catalog No. DM-X6930, Duma Biotechnology Co., Ltd., Shanghai, China) had a detection range of 0–500 μmol/L, with intra-assay variability < 8% and interassay variability < 10%. Serum and CSF samples were thawed at 4 °C, diluted according to the manufacturer’s instructions, and assayed in triplicate. Optical density was measured at 450 nm using a microplate reader (SpectraMax iD3, Molecular Devices, Sunnyvale, CA, USA), and concentrations were calculated from standard curves.

### RT-qPCR Analysis

Total RNA was extracted from spinal cord tissue using TRIzol reagent [29]. A total of 1000 ng of total RNA was reverse-transcribed into cDNA suitable for qPCR analysis using HiScript III All-in-One RT SuperMix Perfect for qPCR (Vazyme Biotech Co., Ltd., Nanjing, China; Cat: R333-01), according to the manufacturer’s protocol. Subsequent RT-qPCR amplification was performed using 1 µg of cDNA template with Taq Pro Universal SYBR qPCR Master Mix (Vazyme Biotech Co., Ltd., Nanjing, China; Cat: Q712-02), following the manufacturer’s instructions. β-Actin was used as the internal reference gene, and the mRNA expression levels of SREBF1, Tlr4, and Myd88 were determined using the SYBR Green method. Gene-specific primers were used for amplification, and all primer sequences are listed in Table S3. The thermal cycling conditions were as follows: initial predenaturation at 95 °C for 30 s, followed by 40 cycles of denaturation at 95 °C for 5 s and annealing/extension at 60 °C for 30 s. The relative mRNA expression levels were calculated using the 2^−ΔΔCt^ method. Three biological replicates were included in each group, and three technical replicates were performed for each sample.

### Western Blot Analysis

Proteins were extracted from spinal cord tissues using RIPA lysis buffer supplemented with protease inhibitor (1:100; Wuhan Servicebio Technology Co., Ltd., Wuhan, Hubei, China). Protein concentrations were quantified using a BCA assay kit (Wuhan Servicebio Technology Co., Ltd., Wuhan, Hubei, China; Cat: G2026-200 T). Equal amounts of protein (30 μg per lane) were separated by SDS-PAGE and transferred onto PVDF membranes (MilliporeSigma, Burlington, MA, USA). The membranes were blocked with 5% skim milk for 2 h at room temperature and then incubated overnight at 4 °C with primary antibodies. Detailed information on the primary antibodies is provided in Table S4. After washing with Tris-buffered saline containing 0.1% Tween-20 (TBST), the membranes were incubated with HRP-conjugated secondary antibodies for 1 h at room temperature. Protein bands were visualized using an ECL chemiluminescence reagent (Shanghai Epizyme Biomedical Technology Co., Ltd., Shanghai, China; Cat: SQ202). Images were acquired using a UVP multifunctional imaging system (UVP, LLC, Upland, CA, USA). Band intensities were quantified using ImageJ v1.54f software (National Institutes of Health, Bethesda, MD, USA), and relative protein levels were normalized to α-tubulin [30]. Three animals were included in each group, and independent replicates were performed in triplicate for each sample.

### Immunofluorescence Staining

Paraffin sections were prepared for immunofluorescence staining. After antigen retrieval and blocking of endogenous peroxidase activity and nonspecific binding, sections were incubated with diluted primary antibodies against CD68, iNOS, and arginase-1 (Table S5) overnight at 4 °C [31]. After washing with PBS, the sections were incubated with the corresponding secondary antibodies for 30 min at room temperature in the dark, followed by incubation with TSA fluorescent dye for 10 min at room temperature in the dark [32]. After antibody elution, these steps were repeated to complete multiplex staining with the primary antibodies. The sections were then counterstained with DAPI to label cell nuclei and mounted with antifade mounting medium. Fluorescence images were acquired using a laser scanning confocal microscope. Three animals from each group were included in the analysis. For each animal, one representative section containing the lesion area was selected, and one field of view (200 ×) at the same location in the lesion center was chosen. The iNOS- and Arg-1-associated fluorescence intensities were quantified using ImageJ v1.54f software (National Institutes of Health, Bethesda, MD, USA), and the iNOS/Arg-1 fluorescence-intensity ratio was calculated.

### TUNEL Assay

Paraffin-embedded sections were deparaffinized, rehydrated, and treated with proteinase K. Sections were then incubated with TUNEL reaction mixture (TdT enzyme: fluorescent labeling solution = 1:9) at 37 °C for 60 min [33]. After mounting with DAPI-containing antifade medium, images were acquired using a fluorescence microscope. Three animals from each group were included in the analysis. For each animal, one representative section containing the lesion area was selected, and one high-power field (400 ×) around the lesion center was randomly selected from that section. The proportion of apoptotic cells was calculated using ImageJ v1.54f software (National Institutes of Health, Bethesda, MD, USA) by dividing the number of TUNEL-positive cells by the total number of cells and multiplying by 100%.

### Statistical Analysis

**Fig. 1:**
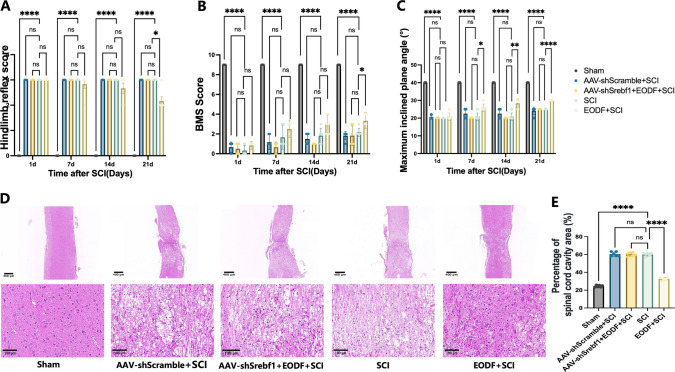
EODF prehabilitation improved functional recovery and reduced histopathological damage after SCI.** A**–**C** Longitudinal assessments of functional recovery in the same cohort of mice from each experimental group on days 1, 7, 14, and 21 after SCI, including **A** Basso Mouse Scale (BMS) locomotor scores, **B** hindlimb reflex scores, and **C** inclined plane test results. Data are presented as mean ± SD; *n* = 6 biologically independent mice per group. **D** Representative hematoxylin and eosin (H&E)-stained transverse spinal cord sections obtained from the region surrounding the lesion epicenter on day 21 after SCI. The upper and lower panels show low- and high-magnification views, respectively. Scale bars, 400 μm in the upper panels and 100 μm in the lower panels. **E** Quantification of the lesion/cavity area, expressed as a percentage of the total spinal cord cross-sectional area. Data are presented as the mean ± SD; *n* = 6 biologically independent mice per group. Longitudinal behavioral data in **A**–**C** were analyzed using two-way repeated-measures ANOVA followed by Dunnett’s multiple-comparisons test. Data in **E** were analyzed using one-way ANOVA followed by Dunnett’s multiple-comparisons test. ^*^p < 0.05, ^**^p < 0.01, and ^****^p < 0.0001; ns, not significant

**Fig. 2:**
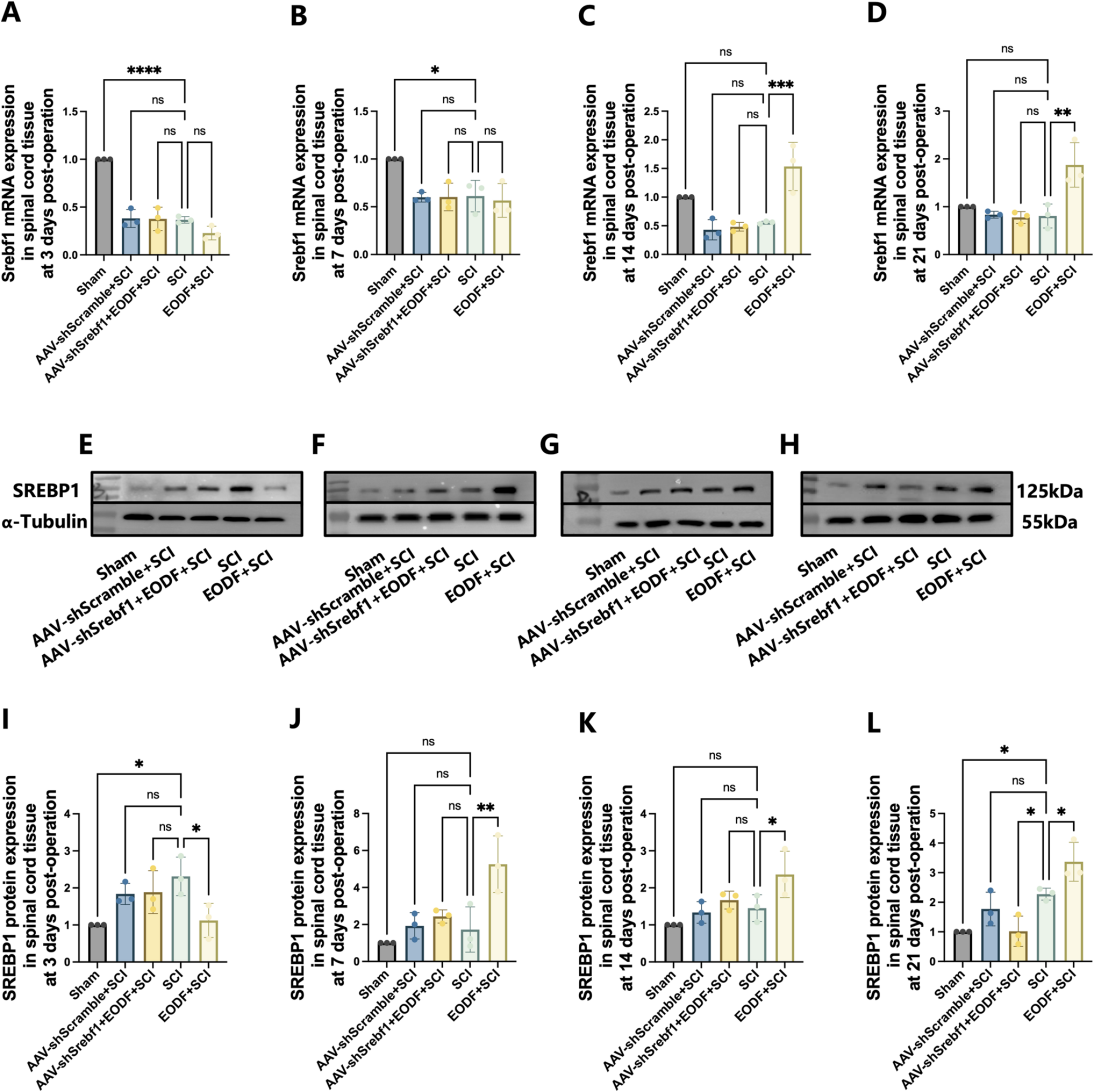
EODF prehabilitation exerted stage-dependent effects onSREBF1 expression after SCI. **A**–**D** Relative SREBF1 mRNA expression in spinal cord tissues collected from the region surrounding the lesion epicenter on days 3, 7, 14, and 21 after SCI, as determined by reverse-transcription quantitative polymerase chain reaction (RT-qPCR). SREBF1 mRNA expression was normalized to β-actin (Actb) and calculated using the 2^−ΔΔCt^ method. **E**–**H** Representative western blot images showing SREBF1 protein expression in spinal cord tissues from each experimental group on days 3, 7, 14, and 21 after SCI, with α-tubulin used as the loading control. **I**–**L** Densitometric quantification of SREBF1 protein expression corresponding to panels **E**–**H**. SREBF1 protein levels were normalized to α-tubulin. Data are presented as the mean ± SD; *n* = 3 biologically independent mice per group at each time point. Statistical comparisons among groups at each time point were performed using one-way ANOVA followed by Dunnett's multiple-comparisons test. ^*^p < 0.05, ^**^p < 0.01, ^***^p < 0.001, and ^****^p < 0.0001; ns, not significant

**Fig. 3:**
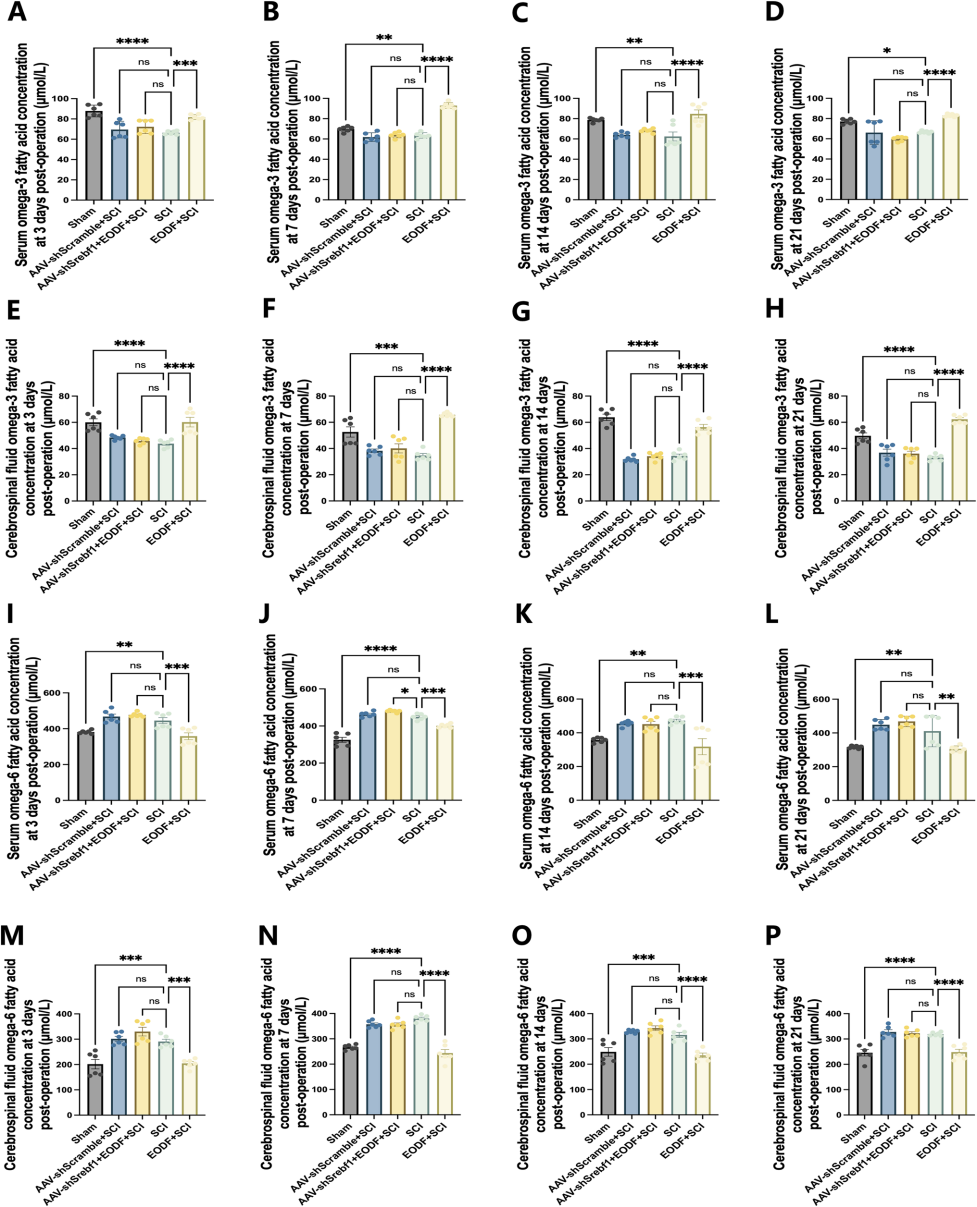
EODF prehabilitation partially restored SCI-associated alterations in omega-3 and omega-6 fatty acid concentrations with the involvement of SREBP1.** A**–**D** Serum omega-3 fatty acid concentrations in each experimental group on days 3, 7, 14, and 21 after SCI, respectively. **E**–**H** Cerebrospinal fluid (CSF) omega-3 fatty acid concentrations in each experimental group at the corresponding time points. **I**–**L** Serum omega-6 fatty acid concentrations in each experimental group on days 3, 7, 14, and 21 after SCI, respectively. **M**–**P** CSF omega-6 fatty acid concentrations in each experimental group at the corresponding time points. Data are presented as mean ± SD; *n* = 6 biologically independent mice per group at each time point. Statistical comparisons among groups at each time point were performed using one-way ANOVA followed by Dunnett’s multiple-comparisons test. ^*^p < 0.05, ^**^p < 0.01**,** ^***^p < 0.001, and ^****^p < 0.0001; ns, not significant

**Fig. 4:**
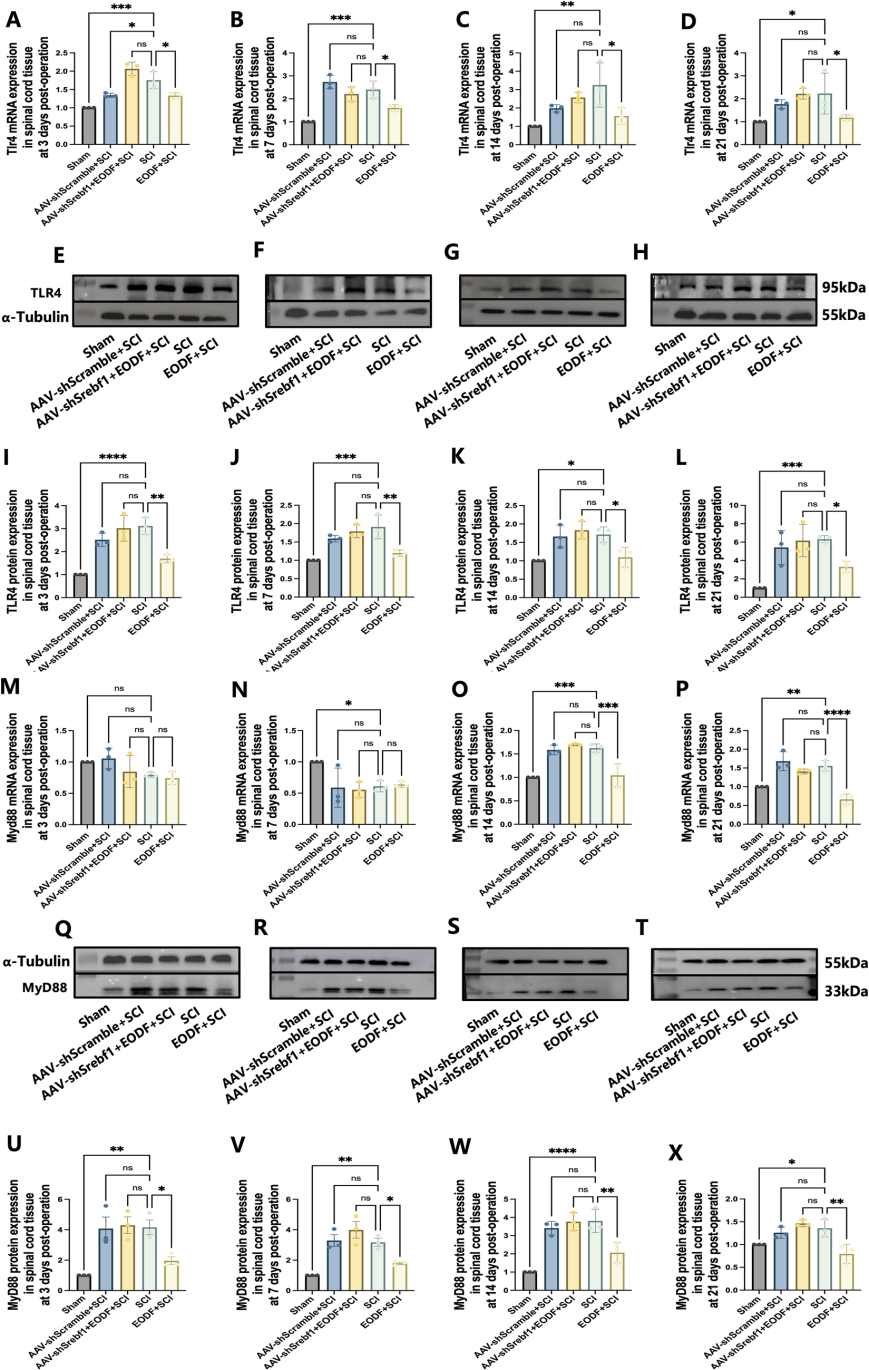
EODF prehabilitation attenuates SCI-associated TLR4/MyD88 signaling with the involvement of SREBP1.** A**–**D** Relative Tlr4 mRNA expression in spinal cord tissues collected from the region surrounding the lesion epicenter on days 3, 7, 14, and 21 after SCI, as determined by reverse-transcription quantitative polymerase chain reaction (RT-qPCR). Tlr4 mRNA expression was normalized to β-actin (Actb) and calculated using the 2^−ΔΔCt^ method. **E**–**H** Representative western blot images showing TLR4 protein expression in spinal cord tissues from each experimental group at the corresponding time points, with α-tubulin used as the loading control. **I**–**L** Densitometric quantification of TLR4 protein expression corresponding to panels **E**–**H**. TLR4 protein levels were normalized to α-tubulin. **M**–**P** Relative Myd88 mRNA expression in spinal cord tissues collected from the region surrounding the lesion epicenter on days 3, 7, 14, and 21 after SCI, as determined by RT-qPCR. Myd88 mRNA expression was normalized to β-actin (Actb) and calculated using the 2^−ΔΔCt^ method. **Q**–**T** Representative western blot images showing MyD88 protein expression in spinal cord tissues from each experimental group at the corresponding time points, with α-tubulin used as the loading control. **U**–**X** Densitometric quantification of MyD88 protein expression corresponding to panels **Q**–**T**. MyD88 protein levels were normalized to α-tubulin. Data are presented as the mean ± SD; *n* = 3 biologically independent mice per group at each time point. Statistical comparisons among groups at each time point were performed using one-way ANOVA followed by Dunnettt’s multiple-comparisons test. ^*^p < 0.05, ^**^p < 0.01**,** ^***^p < 0.001, and ^****^p < 0.0001; ns, not significant

**Fig. 5:**
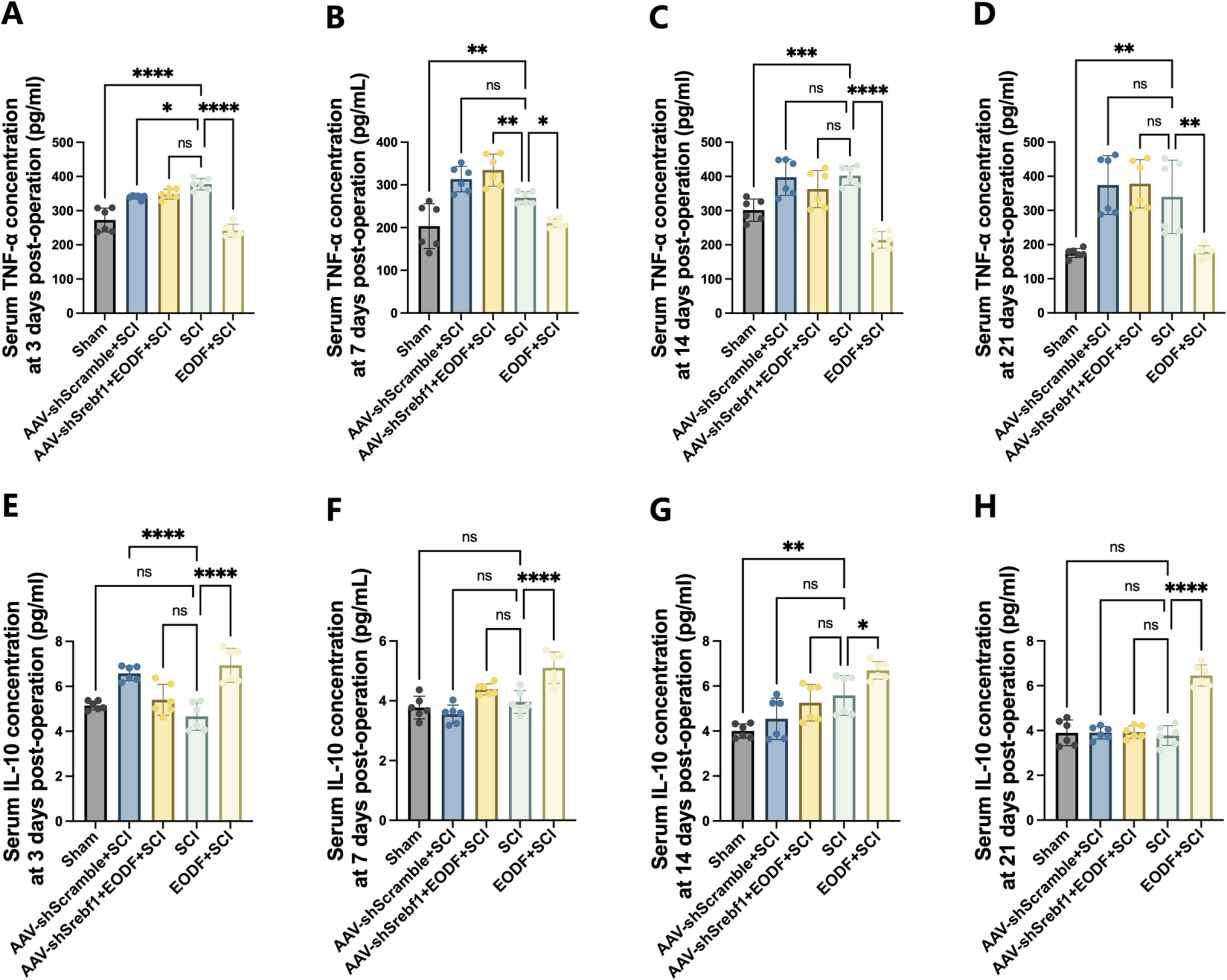
EODF prehabilitation modulated serum inflammatory cytokine levels after SCI with the involvement of SREBP1.** A**–**D** Serum tumor necrosis factor-α (TNF-α) concentrations in each experimental group on days 3, 7, 14, and 21 after SCI, respectively, as determined by enzyme-linked immunosorbent assay (ELISA). **E**–**H** Serum interleukin-10 (IL-10) concentrations in each experimental group at the corresponding time points, as determined by ELISA. Data are presented as the mean ± SD; *n* = 6 biologically independent mice per group at each time point. Statistical comparisons among groups at each time point were performed using one-way ANOVA followed by Dunnett's multiple-comparisons test. ^*^p < 0.05, ^**^p < 0.01**,** ^***^p < 0.001, and ^****^p < 0.0001; ns, not significant

**Fig. 6:**
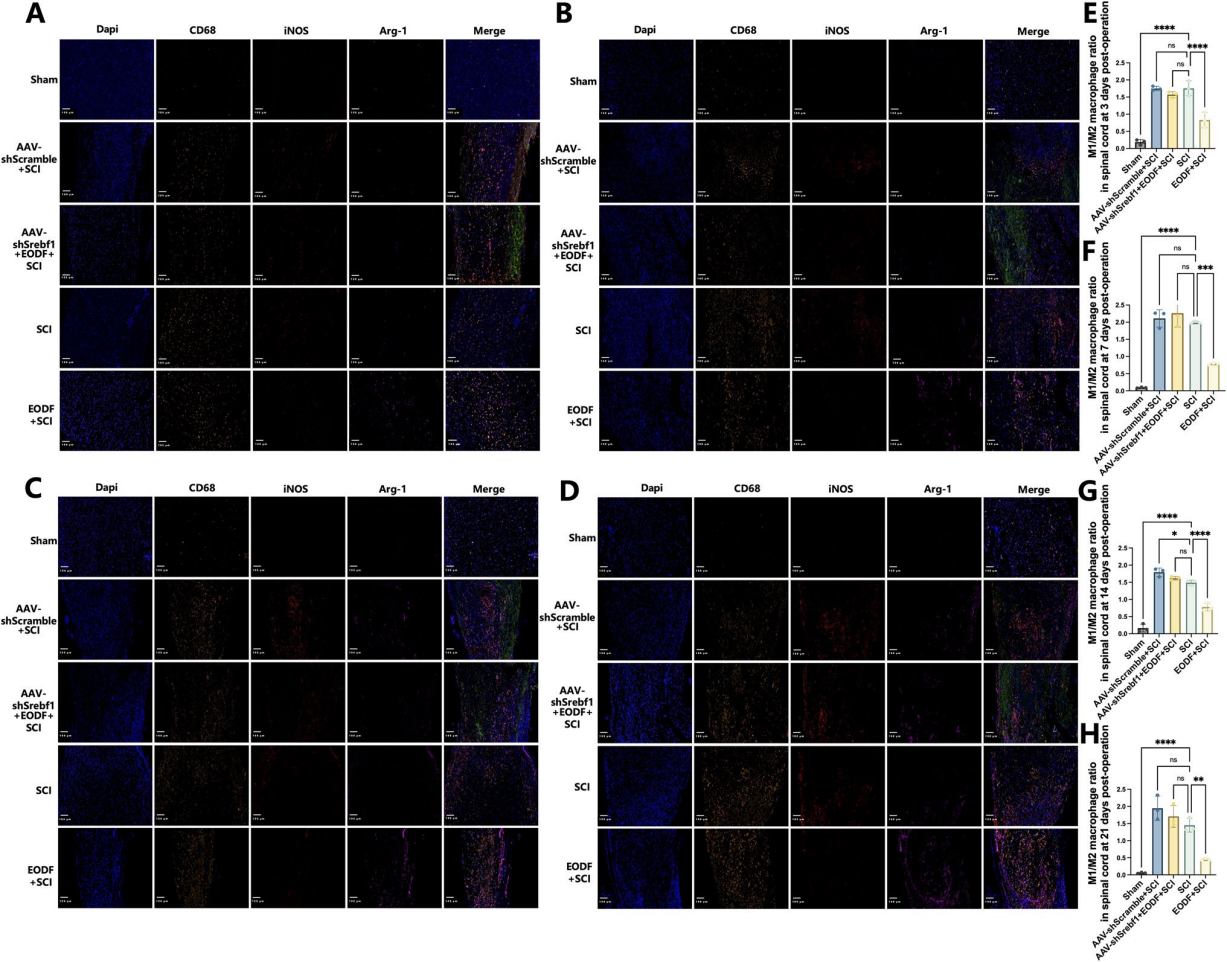
EODF prehabilitation shifted macrophage/microglia-associated markers toward a less proinflammatory profile after SCI with the involvement of SREBF1. **A–D** Representative immunofluorescence images of transverse spinal cord sections collected from the region surrounding the lesion epicenter on days 3, 7, 14, and 21 after SCI, respectively, showing DAPI (blue, nuclei), CD68 (orange, macrophages/microglia), inducible nitric oxide synthase (iNOS; red, M1-associated macrophages/microglia), arginase-1 (Arg-1; magenta, M2-associated macrophages/microglia), and merged images. Scale bars, 100 μm. **E–H** Quantification of the ratio of iNOS-associated fluorescence intensity to Arg-1-associated fluorescence intensity at the corresponding time points. Data are presented as the mean ± SD; *n* = 3 biologically independent mice per group at each time point. Statistical comparisons among groups at each time point were performed using one-way ANOVA followed by Dunnett’s multiple-comparisons test. ^*^p < 0.05, ^**^p < 0.01**,** ^***^p < 0.001, and ^****^p < 0.0001; ns, not significant

**Fig. 7:**
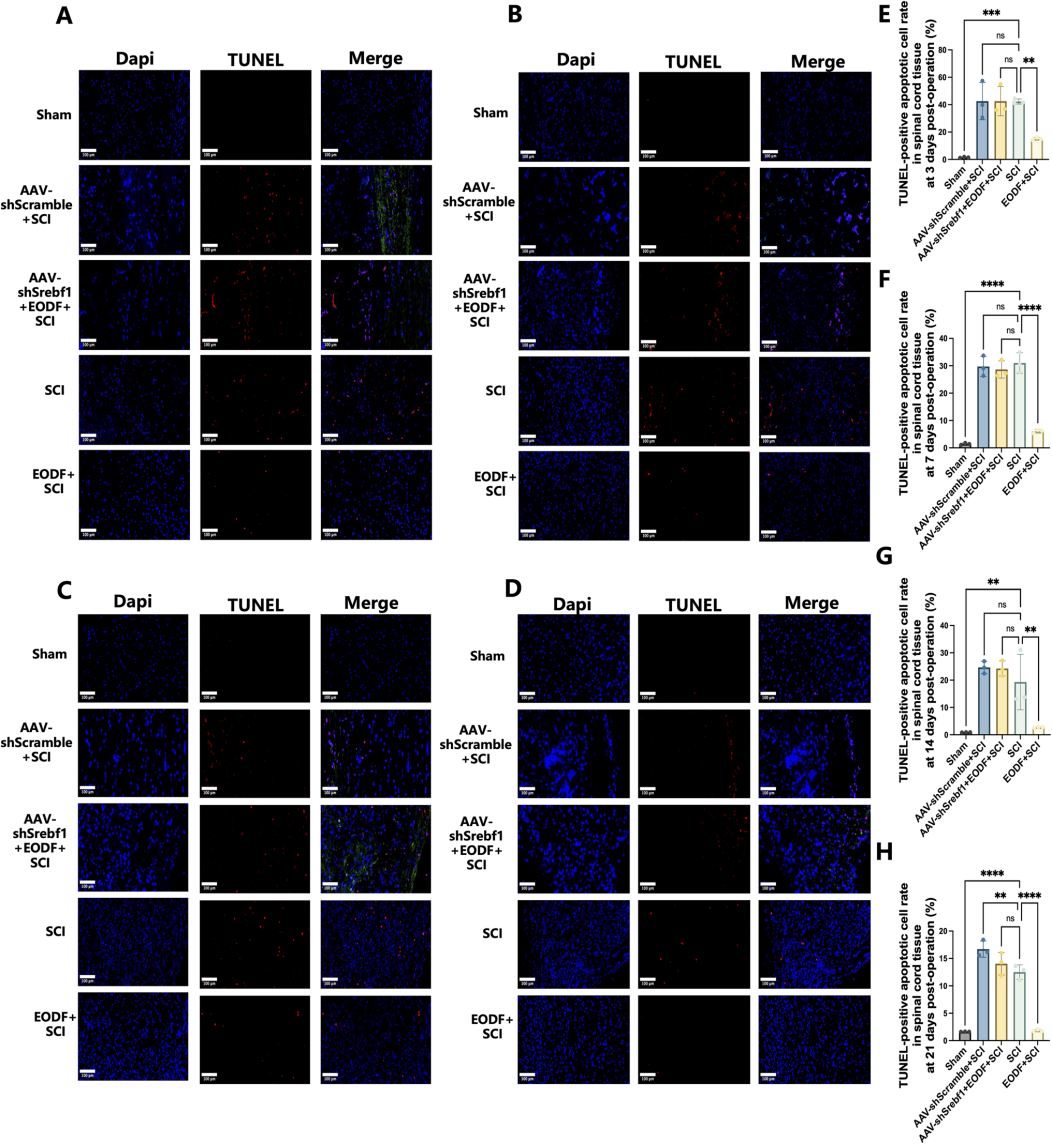
EODF prehabilitation reduced the propotion of TUNEL-positive cells in injured spinal cord tissue after SCI with the involvement of SREBP1.** A**–**D** Representative terminal deoxynucleotidyl transferase dUTP nick end labeling (TUNEL) staining images of transverse spinal cord sections collected from the region surrounding the lesion epicenter on days 3, 7, 14, and 21 after SCI, respectively, showing DAPI (blue, nuclei), TUNEL (red, apoptotic cells), and merged images. Scale bars, 100 μm. **E**–**H** Quantification of the percentage of TUNEL-positive cells at the corresponding time points. Data are presented as the mean ± SD; *n* = 3 biologically independent mice per group at each time point. Statistical comparisons among groups at each time point were performed using one-way ANOVA followed by Dunnett’s multiple-comparisons test. ^*^p < 0.05, ^**^p < 0.01**,** ^***^p < 0.001, and ^****^p < 0.0001; ns, not significant

All experimental data were analyzed using GraphPad Prism 10 software (GraphPad Software, San Diego, CA, USA). Quantitative data are presented as the mean ± SD. Behavioral data obtained from the same animals at multiple time points, including BMS scores, hindlimb reflex scores, and inclined plane performance, were analyzed using repeated-measures two-way ANOVA to evaluate the effects of group, time, and their interaction. Histological, molecular biology, and biochemical data obtained from independent samples across groups were analyzed using one-way or two-way ANOVA, as appropriate for the experimental design. When significant differences were detected, appropriate post hoc multiple-comparison tests were used for pairwise group comparisons. Data that did not meet the assumptions of normality or homogeneity of variance were analyzed using the corresponding nonparametric tests. A value of *p* < 0.05 was considered statistically significant. Exact adjusted *p* values for the prespecified pairwise comparisons across Figs. 1, 2, 3, 4, 5, 6, and 7 are provided in Supplementary Table S6.

## Results

### EODF Prehabilitation Was Well Tolerated in Mice

To evaluate the safety and tolerability of the 4-week EODF prehabilitation regimen, baseline body weight, changes in body weight over time, and daily food intake were monitored throughout the intervention. Before EODF, baseline body weight was comparable across the five experimental groups, with no significant differences among groups (*p* > 0.05; Figure S3A). During the 4-week EODF intervention, mice in the EODF + SCI and AAV-shSrebf1 + EODF + SCI groups exhibited alternating fluctuations in body weight, with decreases on fasting days and recovery on refeeding days (Figure S3B). Daily food intake likewise followed an alternating pattern in the EODF-treated groups, with zero food intake on fasting days and compensatory increases on refeeding days (Figure S3C). Body weight remained within an acceptable range, and no mouse reached the predefined exclusion threshold of 20% body weight loss. No overt adverse effects were observed during the intervention period. These results indicate that the EODF prehabilitation protocol was well tolerated under the present experimental conditions.

### EODF Prehabilitation Improved Functional Recovery and Alleviated Histopathological Damage After SCI

We next examined whether EODF prehabilitation affected neurological recovery after SCI. On postoperative day 1, all injured groups showed severe motor dysfunction, with BMS scores predominantly ranging from 0 to 1 and hindlimb reflex scores of 3; no significant differences were detected among the groups (*p* > 0.05). These findings were consistent with successful establishment of the SCI model and comparable initial injury severity. During recovery, compared with the SCI group, the EODF + SCI group showed gradual functional improvement, including higher BMS scores on postoperative day 21, lower hindlimb reflex scores on postoperative days 14 and 21, and better inclined plane performance from postoperative day 7 onward (*p* < 0.05 to *p* < 0.0001; Fig. 1A–C).

Histological assessment on postoperative day 21 further showed marked tissue disruption, inflammatory cell infiltration, and cavity formation in the SCI and AAV-shScramble + SCI groups (Fig. 1D and E). EODF prehabilitation significantly reduced tissue damage, inflammatory cell infiltration, and lesion cavity area compared with the SCI group (*p* < 0.0001). No significant differences were detected between the SCI and AAV-shScramble + SCI groups in either the behavioral or histological analyses, suggesting no detectable nonspecific effects of the control AAV vector. However, these functional and histological protective effects were markedly weakened in the AAV-shSrebf1 + EODF + SCI group. Overall, these results indicate that EODF prehabilitation improved motor recovery and reduced histopathological damage after SCI and that these effects were markedly diminished after SREBF1 knockdown.

## EODF Prehabilitation Had Stage-Dependent Effects on SREBP1 Expression After SCI

We examined SREBF1 expression at different time points after SCI. RT-qPCR analysis showed that SREBF1 mRNA levels were significantly decreased in all injured groups on postoperative day 3, with no significant differences among them (Fig. 2A and B). At later time points, SREBF1 mRNA expression increased in the EODF + SCI group and was significantly higher than in the SCI and AAV-shSrebf1 + EODF + SCI groups on postoperative days 14 and 21 (*p* < 0.01 to *p* < 0.001; Fig. 2C and D).

Western blot analysis revealed a distinct temporal pattern at the protein level. On postoperative day 3, SREBP1 protein expression was significantly increased in the SCI group compared with the sham group, whereas this increase was attenuated in the EODF + SCI group (*p* < 0.05; Fig. 2I). In contrast, on postoperative days 7, 14, and 21, SREBP1 protein expression was significantly higher in the EODF + SCI group than in the SCI and AAV-shSrebf1 + EODF + SCI groups (*p* < 0.05 to *p* < 0.01; Fig. 2J–L). In the SREBF1 knockdown group, SREBP1 expression remained low across all time points. These results indicate that the effects of EODF prehabilitation on SREBP1 expression were stage dependent, with attenuation of its acute increase and higher expression during later phases.

### EODF Prehabilitation Partially Restored SCI-Associated Fatty Acid Imbalance in Association with SREBP1

To determine whether EODF prehabilitation influenced fatty acid profiles after SCI, omega-3 and omega-6 fatty acid levels were measured in serum and CSF at multiple time points (Fig. 3). Compared with the sham group, the SCI group had significantly lower omega-3 levels and significantly higher omega-6 levels in both serum and CSF throughout the acute, subacute, and chronic stages after injury, This pattern indicated persistent alterations in fatty acid profiles after SCI. EODF prehabilitation partially reversed these changes. In the EODF + SCI group, omega-3 levels were increased and omega-6 levels were decreased relative to those in the SCI group, with similar patterns in serum and CSF. In contrast, these effects were not maintained in the AAV-shSrebf1 + EODF + SCI group, in which omega-3 and omega-6 levels were comparable to those in the SCI group and significantly different from those in the EODF + SCI group. These findings indicate that EODF partially restored SCI-associated alterations in fatty acid profiles and that these effects were diminished by SREBF1 knockdown.

### EODF Prehabilitation Attenuated TLR4/MyD88 Signaling and Modulated Inflammatory Cytokine Levels After SCI

To assess whether EODF prehabilitation affected inflammatory signaling after SCI, we measured TLR4 and MyD88 expression at the mRNA and protein levels (Fig. 4). Compared with the sham group, the SCI group exhibited sustained increases in Tlr4 and Myd88 expression after injury (*p* < 0.05 to *p* < 0.0001; Fig. 4A–D and I–L). EODF prehabilitation attenuated these changes. Tlr4 mRNA expression was significantly lower in the EODF + SCI group than in the SCI group at multiple postinjury time points (*p* < 0.05; Fig. 4A–D). Myd88 mRNA expression was significantly lower in the EODF + SCI group than in the SCI group at later stages, particularly on days 14 and 21 (*p* < 0.001 to *p* < 0.0001; Fig. 4O and P). Consistent with these findings, western blot analysis showed significantly lower TLR4 and MyD88 protein levels in the EODF + SCI group than in the SCI group (*p* < 0.05 to *p* < 0.01; Fig. 4I–L and U–X).

We next evaluated circulating inflammatory cytokines (Fig. 5). ELISA showed that serum TNF-α levels were significantly lower in the EODF + SCI group than in the SCI group at all examined time points (*p* < 0.05 to *p* < 0.0001; Fig. 5A–D), whereas IL-10 levels were significantly higher in the EODF + SCI group (*p* < 0.05 to *p* < 0.0001; Fig. 5E–H). In contrast, these EODF-associated changes in TLR4/MyD88 expression and cytokine levels were largely absent in the AAV-shSrebf1 + EODF + SCI group, whose values were generally comparable to those of the SCI group (Figs. 4 and 5). Overall, EODF prehabilitation attenuated TLR4/MyD88 signaling, reduced TNF-α levels, and increased IL-10 levels after SCI; these effects were markedly reduced by SREBF1 knockdown.

### EODF Prehabilitation Shifted Macrophage/Microglia-Associated Markers Toward a Less Proinflammatory Profile After SCI

To evaluate the effects of EODF prehabilitation on the postinjury immune microenvironment, immunofluorescence staining was performed for CD68 (a pan-macrophage/microglia marker), iNOS (an M1 proinflammatory phenotype marker), and Arg-1 (an M2 anti-inflammatory phenotype marker). The ratio of iNOS-associated to Arg-1-associated fluorescence was used as an index of macrophage/microglial polarization (Fig. 6). Compared with the sham group, the SCI group showed a marked increase in the iNOS/Arg-1-associated fluorescence ratio at all examined time points, consistent with a more proinflammatory marker profile after injury (*p* < 0.01 to *p* < 0.0001; Fig. 6E–H). EODF prehabilitation significantly reduced this ratio at each time point, consistent with a less pro-inflammatory macrophage/microglia marker profile (*p* < 0.05 to *p* < 0.0001; Fig. 6E–H). In contrast, the AAV-shSrebf1 + EODF + SCI group did not show this EODF-associated change and remained comparable to the SCI group across all time points. These findings indicate that EODF altered macrophage/microglia-associated phenotypic markers after SCI and that this effect was markedly diminished by SREBF1 knockdown.

### EODF Prehabilitation Reduced the Proportion of TUNEL-Positive Cells in Injured Spinal Cord Tissue After SCI

We examined whether EODF prehabilitation affected apoptotic cell death after SCI using TUNEL staining (Fig. 7). Compared with the sham group, the SCI group showed a marked increase in TUNEL-positive cells at all assessed time points (*p* < 0.01 to *p* < 0.0001; Fig. 7E–H). EODF prehabilitation significantly reduced the proportion of TUNEL-positive cells at each time point (*p* < 0.05 to *p* < 0.0001; Fig. 7E–H). In the AAV-shSrebf1 + EODF + SCI group, the EODF-associated reduction was not observed, and the proportion of TUNEL-positive cells remained comparable to that in the SCI group. These findings indicate that EODF reduced the proportion of TUNEL-positive cells in injured spinal cord tissue after SCI and that this effect was markedly diminished by SREBF1 knockdown.

## Discussion

In this study, EODF prehabilitation was associated with improved functional and histopathological outcomes after SCI and with coordinated changes across metabolic, inflammatory, and cell injury measures. Rather than representing a series of independent effects, the findings support an integrated model in which EODF modifies the metabolic context of secondary injury, alters the temporal regulation of SREBF1, and is accompanied by a less inflammatory tissue environment. Specifically, EODF improved locomotor recovery and reduced histopathological damage, changes that were accompanied by a partial restoration of fatty acid profiles, attenuation of TLR4/MyD88 signaling, and a shift in macrophage/microglia-associated markers toward a less proinflammatory profile. The weakening of these protective effects after SREBF1 knockdown supports a functionally relevant role for SREBF1 in coordinating this integrated response, although it does not establish SREBF1 as the sole mediator.

Secondary injury after SCI reflects reciprocal interactions among metabolic disturbances, oxidative stress, innate immune activation, and progressive tissue degeneration [34, 35]. Within this network, EODF may act as a preconditioning stimulus rather than as a single-pathway intervention. Dietary prehabilitation can induce adaptive responses before injury and may thereby alter the metabolic environment in which postinjury inflammation develops [36–39]. The stage-dependent SREBF1 pattern observed here is consistent with this interpretation. During the acute phase, SCI increased SREBF1 protein without a corresponding increase in SREBF1 mRNA, whereas EODF attenuated the protein increase. Because SREBF1 activity is controlled by intracellular trafficking, proteolytic processing, and nuclear translocation in addition to transcription [40–43], the early response may reflect posttranscriptional or posttranslational regulation. At later stages, the higher SREBF1 mRNA and SREBP1 protein levels in the EODF group coincided with improved metabolic and inflammatory indices. Thus, the apparently different early and late responses may represent temporal control of SREBF1 rather than a simple beneficial or harmful effect of uniformly increasing or decreasing its expression. Excessive acute activation may contribute to lipid stress, whereas appropriately regulated SREBF1 activity during later phases may support membrane turnover, lipid homeostasis, and tissue remodeling [44, 45]. Direct measurements of SREBF1 activation are required to test this interpretation.

The lipid, inflammatory, and cellular findings can therefore be considered along a common metabolic–immune axis. Omega-3 and omega-6 fatty acids influence membrane composition, lipid mediator production, inflammatory tone, and immune cell behavior [46, 47], while lipid disturbance can amplify innate immune signaling through pathways that include TLR4/MyD88 [48–52]. In the present study, partial normalization of fatty acid profiles occurred in parallel with reduced TLR4/MyD88 expression, lower TNF-alpha, higher IL-10, and a lower iNOS/Arg-1 fluorescence-intensity ratio. These concurrent changes do not prove a linear causal sequence, but they are consistent with reduced metabolic–inflammatory coupling after EODF. This interpretation is also biologically plausible because pro-inflammatory macrophage and microglial states are commonly associated with glycolytic and mitochondrial stress, whereas less inflammatory or reparative programs depend more strongly on oxidative metabolism and fatty acid utilization [53–56]. By moderating this interconnected environment, EODF may reduce conditions that favor secondary neuronal and glial injury, which would provide a unifying explanation for the lower proportion of TUNEL-positive cells, greater tissue preservation, and improved motor recovery.

The intermittent fasting-refeeding pattern may be important for this integrated response. Unlike continuous nutrient restriction, EODF repeatedly changes substrate availability, hormonal signaling, and transcriptional regulation [57]. These oscillations may increase metabolic flexibility and alter the timing and magnitude of SREBP1-related responses to subsequent injury. More broadly, recent SCI studies indicate that metabolic or mitochondrial interventions can converge on common inflammatory checkpoints: MC4R activation preserves mitochondrial homeostasis and suppresses AIM2 inflammasome activation [58], SGK1 inhibition promotes microglial mitophagy and limits AIM2-related pyroptosis [59], and modulation of the inflammatory microenvironment through biomaterial-based delivery or autophagy activation can reduce regulated cell death and improve recovery [60, 61]. Considered together, these observations support the concept that metabolic adaptation, innate immune signaling, and cell survival are mechanistically linked during secondary SCI. The present findings extend this concept to preinjury EODF and identify SREBP1-associated regulation as one component of that response.

The knockdown results strengthen, but do not complete, the mechanistic argument. SREBF1 knockdown attenuated EODF-associated changes across functional, histological, metabolic, inflammatory, and TUNEL outcomes, suggesting that SREBP1 is positioned upstream of several coordinated effects or is required for their maintenance. However, the experimental design did not include an AAV-shScramble + EODF + SCI group or an AAV-shSrebf1 + SCI group without EODF. Consequently, the study cannot fully separate the effects of the viral procedure, SREBF1 knockdown itself, and their interaction with EODF. In addition, knockdown does not distinguish direct SREBP1-dependent effects from secondary compensatory changes, and no rescue or re-expression experiment was performed. The results should therefore be interpreted as evidence of functional involvement rather than proof of a complete SREBP1-centered causal pathway.

Several additional limitations should be considered. No pair-fed control group was included, so the effects of fasting periodicity cannot be fully separated from differences in total energy intake, body weight dynamics, or feeding behavior; an EODF-treated sham group would also help distinguish injury-specific effects from general physiological adaptation. SREBP1 cleavage, nuclear localization, transcriptional activity, and downstream lipogenic targets were not directly measured. Fatty acid assessment was limited to ELISA-based measurements of total omega-3 and omega-6 levels and did not identify individual lipid species, bioactive lipid mediators, endogenous TLR4 ligands, or lipid peroxidation products. The immunofluorescence analysis relied on CD68, iNOS, and Arg-1 and did not distinguish resident microglia from infiltrating monocyte-derived macrophages; accordingly, the iNOS/Arg-1 fluorescence-intensity ratio should be interpreted as a marker-based index of the inflammatory profile rather than as a direct M1/M2 cell ratio. TUNEL staining likewise does not identify the affected cell type or distinguish apoptosis from all other forms of regulated cell death. Finally, only female mice were studied, the sample size for molecular and imaging analyses was limited, and the preinjury design and murine model restrict direct extrapolation to unpredictable traumatic SCI in humans.

Future studies should incorporate the missing viral and dietary control groups together with SREBP1 rescue or re-expression experiments. Stage-specific assessment of SREBP1 cleavage, nuclear localization, transcriptional activity, and downstream targets would clarify whether its acute and later functions differ. Targeted lipidomics, metabolomics, cell-specific manipulation, and single-cell or spatial analyses could define the lipid species, immune cell populations, and tissue compartments involved. Studies in both sexes, additional SCI models, and larger animals are also needed. Clinically, EODF prehabilitation may be most relevant when spinal cord risk can be anticipated, such as selected high-risk spinal procedures or interventions associated with transient spinal cord ischemia. Because repeated fasting may be unsuitable for patients with malnutrition, frailty, diabetes, impaired glucose regulation, or increased perioperative nutritional requirements, translational work should determine safe regimens and evaluate fasting-mimetic approaches that reproduce the metabolic effects without requiring strict dietary restriction.

In conclusion, EODF prehabilitation was associated with greater resistance to SCI-related secondary injury and with coordinated stage-dependent metabolic–immune remodeling. The collective pattern—temporal regulation of SREBF1, partial restoration of fatty acid profiles, attenuation of TLR4/MyD88-associated inflammation, a less proinflammatory marker profile, and reduced TUNEL positivity—supports an integrated mechanism rather than a set of isolated effects. The attenuation of these responses after SREBF1 knockdown indicates that SREBF1 is functionally relevant, while the study design and mechanistic limitations warrant cautious interpretation. These findings provide a rationale for further investigation of temporally controlled metabolic prehabilitation and SREBF1-associated pathways as strategies for enhancing spinal cord resilience.

## Supplementary Information

Below is the link to the electronic supplementary material.

ESM 1 (DOCX 2.12 MB)

## Data Availability

All data supporting the findings of this study are available within the paper and its Supplementary Information.
