# Supplementary material for: Every-Other-Day Fasting Prehabilitation Attenuates Secondary Injury Following Spinal Cord Injury and Is Associated with Stage-Dependent SREBP1-Related Metabolic–Immune Remodeling
Source: Mol Neurobiol. 2026 Aug 17;63(1):842. doi: 10.1007/s12035-026-06139-6 (PMC13481423; doi:10.1007/s12035-026-06139-6)
Supplement: Supplementary file 1 [file 12035_2026_6139_MOESM1_ESM.docx]

**Supplementary Tables**

**Table S1. Label-declared composition of the maintenance chow used in this study**

| **Category** | **Item** | **Description/Value** |
| --- | --- | --- |
| General information | Diet type | Formulated chow for mice and rats |
| General information | Intended use | Maintenance-stage feeding for mice and rats |
| General information | Manufacturer | Shanghai Zhouyu Biotechnology Co., Ltd., Shanghai, China |
| General information | Production license | Hu feed certificate No. (2021) 04027 |
| General information | Product standard | Q/310000ZYSW001-2025 |
| Guaranteed composition | Crude protein | ≥180 g/kg |
| Guaranteed composition | Crude fat | ≥40 g/kg |
| Guaranteed composition | Crude fiber | ≤50 g/kg |
| Guaranteed composition | Crude ash | ≤80 g/kg |
| Guaranteed composition | Lysine | ≥8.2 g/kg |
| Guaranteed composition | Methionine + cystine | ≥5.3 g/kg |
| Guaranteed composition | Vitamin A | ≥7000 IU/kg |
| Guaranteed composition | Vitamin E | ≥60 mg/kg |
| Guaranteed composition | Calcium | 10–18 g/kg |
| Guaranteed composition | Total phosphorus | 6–12 g/kg |
| Guaranteed composition | Moisture | ≤100 g/kg |
| Experimental relevance | Group consistency | The same chow was provided  to all experimental groups |
| Experimental relevance | Dietary intervention  difference | The EODF intervention differed only in feeding schedule  rather than chow composition |

Data were obtained from the product label of the maintenance chow used in this study. The chow was an irradiated formulated feed for mice and rats intended for maintenance-stage feeding. All experimental groups received the same chow; therefore, the EODF intervention differed only in feeding schedule rather than chow composition.

**Table S2. Candidate shRNA sequences and screening efficiency for mouse Srebf1 knockdown**

| **Construct ID** | **Sequence**  **(5′→3′)** | | **Relative**  **expression** | **Knockdown**  **efficiency (%)** | | **Selection** |
| --- | --- | --- | --- | --- | --- | --- |
| BC-1592  shRNA1 | ACTCAGCAAAGTCCCTGATGAC | 697.62 | | 9.76 | — | |
| BC-1593  shRNA2 | ACCTGTGTCTGGTCCATTGCCA | 114.79 | | 85.15 | — | |
| BC-1594  shRNA3 | TTGCTGCTTCTAACCTGGCA | 59.68 | | 92.28 | selected | |
| Scramble  control | CCTAAGGTTAAGTCGCCCTCG | — | | — | Negative  control | |

The knockdown efficiency of candidate shRNAs targeting mouse Srebf1 was evaluated in 293T cells by co-transfection of the Srebf1 overexpression plasmid and individual shRNA plasmids. Total RNA was extracted 48 h after transfection, and Srebf1 expression was quantified by RT-qPCR. BC-1594 showed the highest knockdown efficiency and was selected for AAV packaging.

**Table S3. Primer sequences for RT-qPCR analysis**

| **Gene** | **Primer sequences** | |
| --- | --- | --- |
| Tlr4 | F: ACTCAGCAAAGTCCCTGATGAC | R: GAGGTGGTGTAAGCCATGCCA |
| Myd88 | F: ACCTGTGTCTGGTCCATTGCCA | R: GCTGAGTGCAAACTTGGTCTGG |
| Srebf1 | F: TTGCTGCTTCTAACCTGGCA | R: GAGGCTGGTTTTGACCCTCA |
| β-actin | F: GGCTGTATTCCCCTCCATCG | R: CCAGTTGGTAACAATGCCATGT |

Tlr4: Toll-like receptor 4; Myd88: myeloid differentiation primary response 88; Srebf1: sterol regulatory element-binding transcription factor 1

**Table S4. Primary antibodies for western blot analysis**

| **Antibody** | **Supplier** | **Dilution** | **Source** | **Molecular weight**  **(kDa)** |
| --- | --- | --- | --- | --- |
| TLR4 | Abcam | 1:1000 | Rabbit | 95 |
| MYD88 | Abcam | 1:1000 | Rabbit | 33 |
| SREBP1 | Abcam | 1:1000 | Rabbit | 125 |
| α-Tubulin | Abcam | 1:1000 | Rabbit | 55 |
| GAPDH | Proteintech | 1:1000 | Rabbit | 36 |

TLR4: Toll-like receptor 4; MyD88: myeloid differentiation primary response 88; SREBP1: sterol regulatory element-binding protein 1; GAPDH, glyceraldehyde-3-phosphate dehydrogenase

**Table S5. Antibodies for immunofluorescence staining**

| **Antibody** | **Supplier** | **Dilution** | **Source** |
| --- | --- | --- | --- |
| CD68 | Abcam | 1:200 | Rabbit |
| iNOS | Abcam | 1:200 | Rabbit |
| Arg-1 | Proteintech | 1:200 | Rabbit |

CD 68: cluster of differentiation 68; iNOS: inducible nitric oxide synthase; Arg-1: arginase-1

**Table S6. Summary of selected adjusted P values across Figures 1–7**

Only selected post hoc comparisons are shown: SCI vs AAV-shScramble+SCI, SCI vs EODF+SCI, and EODF+SCI vs AAV-shSrebf1+EODF+SCI. Adjusted P values were obtained from Dunnett’s multiple comparisons test after ordinary one-way ANOVA.

| **Figure / Panel** | **Outcome** | **Comparison** | **Time point** | **Adjusted P value** | **Interpretation** |
| --- | --- | --- | --- | --- | --- |
| Figure 1A | BMS score | SCIvs EODF+SCI | Day 21 | 0.0230 | EODF improved locomotor recovery |
| Figure 1A | BMS score | EODF+SCI vs AAV-shSrebf1+EODF+SCI | Day 7 | 0.0106 | Srebf1 knockdown attenuated EODF-associated improvement |
| Figure 1A | BMS score | EODF+SCI vs AAV-shSrebf1+EODF+SCI | Day 14 | 0.0143 | Srebf1 knockdown attenuated EODF-associated improvement |
| Figure 1A | BMS score | EODF+SCI vs AAV-shSrebf1+EODF+SCI | Day 21 | 0.0096 | Srebf1 knockdown attenuated EODF-associated improvement |
| Figure 1B | Hindlimb reflex score | SCI vs EODF+SCI | Day 21 | 0.0210 | EODF improved hindlimb reflex recovery |
| Figure 1B | Hindlimb reflex score | EODF+SCI vs AAV-shSrebf1+EODF+SCI | Day 21 | 0.0210 | Srebf1 knockdown attenuated EODF-associated improvement |
| Figure 1C | Inclined plane performance | SCI vs EODF+SCI | Day 7 | 0.0116 | EODF improved inclined plane performance |
| Figure 1C | Inclined plane performance | SCI vs EODF+SCI | Day 14 | 0.0020 | EODF improved inclined plane performance |
| Figure 1C | Inclined plane performance | SCI vs EODF+SCI | Day 21 | <0.0001 | EODF improved inclined plane performance |
| Figure 1C | Inclined plane performance | EODF+SCI vs AAV-shSrebf1+EODF+SCI | Day 7 | 0.0049 | Srebf1 knockdown attenuated EODF-associated improvement |
| Figure 1C | Inclined plane performance | EODF+SCI vs AAV-shSrebf1+EODF+SCI | Day 14 | 0.0028 | Srebf1 knockdown attenuated EODF-associated improvement |
| Figure 1C | Inclined plane performance | EODF+SCI vs AAV-shSrebf1+EODF+SCI | Day 21 | <0.0001 | Srebf1 knockdown attenuated EODF-associated improvement |
| Figure 2C | Srebf1 mRNA expression | SCI vs EODF+SCI | Day 14 | 0.0006 | EODF significantly altered Srebf1 mRNA expression |
| Figure 2D | Srebf1 mRNA expression | SCI vs EODF+SCI | Day 21 | 0.0011 | EODF significantly altered Srebf1 mRNA expression |
| Figure 2D | Srebf1 mRNA expression | EODF+SCI vs AAV-shSrebf1+EODF+SCI | Day 21 | 0.0009 | AAV-shSrebf1 significantly altered the EODF-associated change |
| Figure 2I | SREBP1 protein expression | SCI vs EODF+SCI | Day 3 | 0.0204 | EODF significantly altered SREBP1 protein expression |
| Figure 2J | SREBP1 protein expression | SCI vs EODF+SCI | Day 7 | 0.0034 | EODF significantly altered SREBP1 protein expression |
| Figure 2J | SREBP1 protein expression | EODF+SCI vs AAV-shSrebf1+EODF+SCI | Day 7 | 0.0143 | AAV-shSrebf1 significantly altered the EODF-associated change |
| Figure 2K | SREBP1 protein expression | SCI vs EODF+SCI | Day 14 | 0.0386 | EODF significantly altered SREBP1 protein expression |
| Figure 2L | SREBP1 protein expression | SCI vs EODF+SCI | Day 21 | 0.0487 | EODF significantly altered SREBP1 protein expression |
| Figure 2L | SREBP1 protein expression | EODF+SCI vs AAV-shSrebf1+EODF+SCI | Day 21 | 0.0003 | AAV-shSrebf1 significantly altered the EODF-associated change |
| Figure 3A | Serum omega-3 fatty acid concentration | SCI vs EODF+SCI | Day 3 | 0.0002 | EODF significantly increased serum omega-3 fatty acid concentration |
| Figure 3A | Serum omega-3 fatty acid concentration | EODF+SCI vs AAV-shSrebf1+EODF+SCI | Day 3 | 0.0226 | Srebf1 knockdown attenuated the EODF-associated increase |
| Figure 3B | Serum omega-3 fatty acid concentration | SCI vs EODF+SCI | Day 7 | <0.0001 | EODF significantly increased serum omega-3 fatty acid concentration |
| Figure 3B | Serum omega-3 fatty acid concentration | EODF+SCI vs AAV-shSrebf1+EODF+SCI | Day 7 | <0.0001 | Srebf1 knockdown attenuated the EODF-associated increase |
| Figure 3C | Serum omega-3 fatty acid concentration | SCI vs EODF+SCI | Day 14 | <0.0001 | EODF significantly increased serum omega-3 fatty acid concentration |
| Figure 3C | Serum omega-3 fatty acid concentration | EODF+SCI vs AAV-shSrebf1+EODF+SCI | Day 14 | 0.0010 | Srebf1 knockdown attenuated the EODF-associated increase |
| Figure 3D | Serum omega-3 fatty acid concentration | SCI vs EODF+SCI | Day 21 | <0.0001 | EODF significantly increased serum omega-3 fatty acid concentration |
| Figure 3D | Serum omega-3 fatty acid concentration | EODF+SCI vs AAV-shSrebf1+EODF+SCI | Day 21 | <0.0001 | Srebf1 knockdown attenuated the EODF-associated increase |
| Figure 3E | Cerebrospinal fluid omega-3 fatty acid concentration | SCI vs EODF+SCI | Day 3 | <0.0001 | EODF significantly increased cerebrospinal fluid omega-3 fatty acid concentration |
| Figure 3E | Cerebrospinal fluid omega-3 fatty acid concentration | EODF+SCI vs AAV-shSrebf1+EODF+SCI | Day 3 | 0.0003 | Srebf1 knockdown attenuated the EODF-associated increase |
| Figure 3F | Cerebrospinal fluid omega-3 fatty acid concentration | SCI vs EODF+SCI | Day 7 | <0.0001 | EODF significantly increased cerebrospinal fluid omega-3 fatty acid concentration |
| Figure 3F | Cerebrospinal fluid omega-3 fatty acid concentration | EODF+SCI vs AAV-shSrebf1+EODF+SCI | Day 7 | <0.0001 | Srebf1 knockdown attenuated the EODF-associated increase |
| Figure 3G | Cerebrospinal fluid omega-3 fatty acid concentration | SCI vs EODF+SCI | Day 14 | <0.0001 | EODF significantly altered cerebrospinal fluid omega-3 fatty acid concentration |
| Figure 3G | Cerebrospinal fluid omega-3 fatty acid concentration | EODF+SCI vs AAV-shSrebf1+EODF+SCI | Day 14 | <0.0001 | Srebf1 knockdown altered the EODF-associated change |
| Figure 3H | Cerebrospinal fluid omega-3 fatty acid concentration | SCI vs EODF+SCI | Day 21 | <0.0001 | EODF significantly altered cerebrospinal fluid omega-3 fatty acid concentration |
| Figure 3H | Cerebrospinal fluid omega-3 fatty acid concentration | EODF+SCI vs AAV-shSrebf1+EODF+SCI | Day 21 | <0.0001 | Srebf1 knockdown altered the EODF-associated change |
| Figure 3I | Serum omega-6 fatty acid concentration | SCI vs EODF+SCI | Day 3 | 0.0003 | EODF significantly altered serum omega-6 fatty acid concentration |
| Figure 3I | Serum omega-6 fatty acid concentration | EODF+SCI vs AAV-shSrebf1+EODF+SCI | Day 3 | <0.0001 | Srebf1 knockdown altered the EODF-associated change |
| Figure 3J | Serum omega-6 fatty acid concentration | SCI vs EODF+SCI | Day 7 | 0.0004 | EODF significantly altered serum omega-6 fatty acid concentration |
| Figure 3J | Serum omega-6 fatty acid concentration | EODF+SCI vs AAV-shSrebf1+EODF+SCI | Day 7 | <0.0001 | Srebf1 knockdown altered the EODF-associated change |
| Figure 3K | Serum omega-6 fatty acid concentration | SCI vs EODF+SCI | Day 14 | 0.0003 | EODF significantly altered serum omega-6 fatty acid concentration |
| Figure 3K | Serum omega-6 fatty acid concentration | EODF+SCI vs AAV-shSrebf1+EODF+SCI | Day 14 | 0.0015 | Srebf1 knockdown altered the EODF-associated change |
| Figure 3L | Serum omega-6 fatty acid concentration | SCI vs EODF+SCI | Day 21 | 0.0024 | EODF significantly altered serum omega-6 fatty acid concentration |
| Figure 3L | Serum omega-6 fatty acid concentration | EODF+SCI vs AAV-shSrebf1+EODF+SCI | Day 21 | <0.0001 | Srebf1 knockdown altered the EODF-associated change |
| Figure 3M | Cerebrospinal fluid omega-6 fatty acid concentration | SCI vs EODF+SCI | Day 3 | 0.0004 | EODF significantly altered cerebrospinal fluid omega-6 fatty acid concentration |
| Figure 3M | Cerebrospinal fluid omega-6 fatty acid concentration | EODF+SCI vs AAV-shSrebf1+EODF+SCI | Day 3 | <0.0001 | Srebf1 knockdown altered the EODF-associated change |
| Figure 3N | Cerebrospinal fluid omega-6 fatty acid concentration | SCI vs EODF+SCI | Day 7 | <0.0001 | EODF significantly altered cerebrospinal fluid omega-6 fatty acid concentration |
| Figure 3N | Cerebrospinal fluid omega-6 fatty acid concentration | EODF+SCI vs AAV-shSrebf1+EODF+SCI | Day 7 | <0.0001 | Srebf1 knockdown altered the EODF-associated change |
| Figure 3O | Cerebrospinal fluid omega-6 fatty acid concentration | SCI vs EODF+SCI | Day 14 | <0.0001 | EODF significantly altered cerebrospinal fluid omega-6 fatty acid concentration |
| Figure 3O | Cerebrospinal fluid omega-6 fatty acid concentration | EODF+SCI vs AAV-shSrebf1+EODF+SCI | Day 14 | <0.0001 | Srebf1 knockdown altered the EODF-associated change |
| Figure 3P | Cerebrospinal fluid omega-6 fatty acid concentration | SCI vs EODF+SCI | Day 21 | <0.0001 | EODF significantly altered cerebrospinal fluid omega-6 fatty acid concentration |
| Figure 3P | Cerebrospinal fluid omega-6 fatty acid concentration | EODF+SCI vs AAV-shSrebf1+EODF+SCI | Day 21 | <0.0001 | Srebf1 knockdown altered the EODF-associated change |
| Figure 4A | Tlr4 mRNA expression | SCI vs AAV-shScramble+SCI | Day 3 | 0.0151 | AAV-shScramble+SCI differed significantly from SCI |
| Figure 4A | Tlr4 mRNA expression | SCI vs EODF+SCI | Day 3 | 0.0136 | EODF significantly reduced Tlr4 mRNA expression |
| Figure 4A | Tlr4 mRNA expression | EODF+SCI vs AAV-shSrebf1+EODF+SCI | Day 3 | 0.0003 | Srebf1 knockdown attenuated the EODF-associated reduction |
| Figure 4B | Tlr4 mRNA expression | SCI vs EODF+SCI | Day 7 | 0.0115 | EODF significantly reduced Tlr4 mRNA expression |
| Figure 4B | Tlr4 mRNA expression | EODF+SCI vs AAV-shSrebf1+EODF+SCI | Day 7 | 0.0485 | Srebf1 knockdown significantly attenuated the EODF-associated reduction |
| Figure 4C | Tlr4 mRNA expression | SCI vs EODF+SCI | Day 14 | 0.0077 | EODF significantly reduced Tlr4 mRNA expression |
| Figure 4C | Tlr4 mRNA expression | EODF+SCI vs AAV-shSrebf1+EODF+SCI | Day 14 | 0.0419 | Srebf1 knockdown significantly attenuated the EODF-associated reduction |
| Figure 4D | Tlr4 mRNA expression | SCI vs EODF+SCI | Day 21 | 0.0397 | EODF significantly reduced Tlr4 mRNA expression |
| Figure 4D | Tlr4 mRNA expression | EODF+SCI vs AAV-shSrebf1+EODF+SCI | Day 21 | 0.0414 | Srebf1 knockdown attenuated the EODF-associated reduction |
| Figure 4I | TLR4 protein expression | SCI vs EODF+SCI | Day 3 | 0.0014 | EODF significantly reduced TLR4 protein expression |
| Figure 4I | TLR4 protein expression | EODF+SCI vs AAV-shSrebf1+EODF+SCI | Day 3 | 0.0024 | Srebf1 knockdown significantly attenuated the EODF-associated reduction |
| Figure 4J | TLR4 protein expression | SCI vs EODF+SCI | Day 7 | 0.0018 | EODF significantly reduced TLR4 protein expression |
| Figure 4J | TLR4 protein expression | EODF+SCI vs AAV-shSrebf1+EODF+SCI | Day 7 | 0.0063 | Srebf1 knockdown significantly attenuated the EODF-associated reduction |
| Figure 4K | TLR4 protein expression | SCI vs EODF+SCI | Day 14 | 0.0270 | EODF significantly reduced TLR4 protein expression |
| Figure 4K | TLR4 protein expression | EODF+SCI vs AAV-shSrebf1+EODF+SCI | Day 14 | 0.0100 | Srebf1 knockdown significantly attenuated the EODF-associated reduction |
| Figure 4L | TLR4 protein expression | SCI vs EODF+SCI | Day 21 | 0.0340 | EODF significantly reduced TLR4 protein expression |
| Figure 4L | TLR4 protein expression | EODF+SCI vs AAV-shSrebf1+EODF+SCI | Day 21 | 0.0446 | Srebf1 knockdown significantly attenuated the EODF-associated reduction |
| Figure 4O | Myd88 mRNA expression | SCI vs EODF+SCI | Day 14 | 0.0007 | EODF significantly reduced Myd88 mRNA expression |
| Figure 4O | Myd88 mRNA expression | EODF+SCI vs AAV-shSrebf1+EODF+SCI | Day 14 | 0.0003 | Srebf1 knockdown significantly attenuated the EODF-associated reduction |
| Figure 4P | Myd88 mRNA expression | SCI vs EODF+SCI | Day 21 | <0.0001 | EODF significantly reduced Myd88 mRNA expression |
| Figure 4P | Myd88 mRNA expression | EODF+SCI vs AAV-shSrebf1+EODF+SCI | Day 21 | 0.0004 | Srebf1 knockdown significantly attenuated the EODF-associated reduction |
| Figure 4U | MYD88 protein expression | SCI vs EODF+SCI | Day 3 | 0.0292 | EODF significantly reduced MYD88 protein expression |
| Figure 4U | MYD88 protein expression | EODF+SCI vs AAV-shSrebf1+EODF+SCI | Day 3 | 0.0209 | Srebf1 knockdown significantly attenuated the EODF-associated reduction |
| Figure 4V | MYD88 protein expression | SCI vs EODF+SCI | Day 7 | 0.0396 | EODF significantly reduced MYD88 protein expression |
| Figure 4V | MYD88 protein expression | EODF+SCI vs AAV-shSrebf1+EODF+SCI | Day 7 | 0.0024 | Srebf1 knockdown significantly attenuated the EODF-associated reduction |
| Figure 4W | MYD88 protein expression | SCI vs EODF+SCI | Day 14 | 0.0037 | EODF significantly reduced MYD88 protein expression |
| Figure 4W | MYD88 protein expression | EODF+SCI vs AAV-shSrebf1+EODF+SCI | Day 14 | 0.0043 | Srebf1 knockdown significantly attenuated the EODF-associated reduction |
| Figure 4X | MYD88 protein expression | SCI vs EODF+SCI | Day 21 | 0.0024 | EODF significantly reduced MYD88 protein expression |
| Figure 4X | MYD88 protein expression | EODF+SCI vs AAV-shSrebf1+EODF+SCI | Day 21 | 0.0007 | Srebf1 knockdown significantly attenuated the EODF-associated reduction |
| Figure 5A | Serum TNF-α concentration | SCI vs AAV-shScramble+SCI | Day 3 | 0.0455 | AAV-shScramble+SCI differed significantly from SCI |
| Figure 5A | Serum TNF-α concentration | SCI vs EODF+SCI | Day 3 | <0.0001 | EODF significantly reduced serum TNF-α concentration |
| Figure 5A | Serum TNF-α concentration | EODF+SCI vs AAV-shSrebf1+EODF+SCI | Day 3 | <0.0001 | Srebf1 knockdown attenuated the EODF-associated reduction in serum TNF-α |
| Figure 5B | Serum TNF-α concentration | SCI vs AAV-shScramble+SCI | Day 7 | 0.1801 | AAV-shScramble+SCI did not differ significantly from SCI |
| Figure 5B | Serum TNF-α concentration | SCI vs EODF+SCI | Day 7 | 0.0005 | EODF significantly reduced serum TNF-α concentration |
| Figure 5B | Serum TNF-α concentration | EODF+SCI vs AAV-shSrebf1+EODF+SCI | Day 7 | <0.0001 | Srebf1 knockdown attenuated the EODF-associated reduction in serum TNF-α |
| Figure 5C | Serum TNF-α concentration | SCI vs AAV-shScramble+SCI | Day 14 | 0.9988 | AAV-shScramble+SCI did not differ significantly from SCI |
| Figure 5C | Serum TNF-α concentration | SCI vs EODF+SCI | Day 14 | <0.0001 | EODF significantly reduced serum TNF-α concentration |
| Figure 5C | Serum TNF-α concentration | EODF+SCI vs AAV-shSrebf1+EODF+SCI | Day 14 | <0.0001 | Srebf1 knockdown attenuated the EODF-associated reduction in serum TNF-α |
| Figure 5D | Serum TNF-α concentration | SCI vs AAV-shScramble+SCI | Day 21 | 0.8078 | AAV-shScramble+SCI did not differ significantly from SCI |
| Figure 5D | Serum TNF-α concentration | SCI vs EODF+SCI | Day 21 | 0.0018 | EODF significantly reduced serum TNF-α concentration |
| Figure 5D | Serum TNF-α concentration | EODF+SCI vs AAV-shSrebf1+EODF+SCI | Day 21 | 0.0002 | Srebf1 knockdown attenuated the EODF-associated reduction in serum TNF-α |
| Figure 5E | Serum IL-10 concentration | SCI vs AAV-shScramble+SCI | Day 3 | <0.0001 | AAV-shScramble+SCI differed significantly from SCI |
| Figure 5E | Serum IL-10 concentration | SCI vs EODF+SCI | Day 3 | <0.0001 | EODF significantly increased serum IL-10 concentration |
| Figure 5E | Serum IL-10 concentration | EODF+SCI vs AAV-shSrebf1+EODF+SCI | Day 3 | 0.0003 | Srebf1 knockdown attenuated the EODF-associated increase in serum IL-10 |
| Figure 5F | Serum IL-10 concentration | SCI vs AAV-shScramble+SCI | Day 7 | 0.1985 | AAV-shScramble+SCI did not differ significantly from SCI |
| Figure 5F | Serum IL-10 concentration | SCI vs EODF+SCI | Day 7 | <0.0001 | EODF significantly increased serum IL-10 concentration |
| Figure 5F | Serum IL-10 concentration | EODF+SCI vs AAV-shSrebf1+EODF+SCI | Day 7 | 0.0091 | Srebf1 knockdown attenuated the EODF-associated increase in serum IL-10 |
| Figure 5G | Serum IL-10 concentration | SCI vs AAV-shScramble+SCI | Day 14 | 0.0733 | AAV-shScramble+SCI did not differ significantly from SCI |
| Figure 5G | Serum IL-10 concentration | SCI vs EODF+SCI | Day 14 | 0.0074 | EODF significantly increased serum IL-10 concentration |
| Figure 5G | Serum IL-10 concentration | EODF+SCI vs AAV-shSrebf1+EODF+SCI | Day 14 | 0.0016 | Srebf1 knockdown attenuated the EODF-associated increase in serum IL-10 |
| Figure 5H | Serum IL-10 concentration | SCI vs AAV-shScramble+SCI | Day 21 | 0.9609 | AAV-shScramble+SCI did not differ significantly from SCI |
| Figure 5H | Serum IL-10 concentration | SCI vs EODF+SCI | Day 21 | <0.0001 | EODF significantly increased serum IL-10 concentration |
| Figure 5H | Serum IL-10 concentration | EODF+SCI vs AAV-shSrebf1+EODF+SCI | Day 21 | <0.0001 | Srebf1 knockdown attenuated the EODF-associated increase in serum IL-10 |
| Figure 6E | M1/M2 macrophage ratio in spinal cord tissue | SCI vs AAV-shScramble+SCI | Day 3 | >0.9999 | AAV-shScramble+SCI did not differ significantly from SCI |
| Figure 6E | M1/M2 macrophage ratio in spinal cord tissue | SCI vs EODF+SCI | Day 3 | <0.0001 | EODF significantly reduced the M1/M2 macrophage ratio |
| Figure 6E | M1/M2 macrophage ratio in spinal cord tissue | EODF+SCI vs AAV-shSrebf1+EODF+SCI | Day 3 | 0.0005 | Srebf1 knockdown attenuated the EODF-associated reduction in the M1/M2 macrophage ratio |
| Figure 6F | M1/M2 macrophage ratio in spinal cord tissue | SCI vs AAV-shScramble+SCI | Day 7 | 0.8849 | AAV-shScramble+SCI did not differ significantly from SCI |
| Figure 6F | M1/M2 macrophage ratio in spinal cord tissue | SCI vs EODF+SCI | Day 7 | 0.0002 | EODF significantly reduced the M1/M2 macrophage ratio |
| Figure 6F | M1/M2 macrophage ratio in spinal cord tissue | EODF+SCI vs AAV-shSrebf1+EODF+SCI | Day 7 | <0.0001 | Srebf1 knockdown attenuated the EODF-associated reduction in the M1/M2 macrophage ratio |
| Figure 6G | M1/M2 macrophage ratio in spinal cord tissue | SCI vs AAV-shScramble+SCI | Day 14 | 0.0122 | AAV-shScramble+SCI differed significantly from SCI |
| Figure 6G | M1/M2 macrophage ratio in spinal cord tissue | SCI vs EODF+SCI | Day 14 | <0.0001 | EODF significantly reduced the M1/M2 macrophage ratio |
| Figure 6G | M1/M2 macrophage ratio in spinal cord tissue | EODF+SCI vs AAV-shSrebf1+EODF+SCI | Day 14 | <0.0001 | Srebf1 knockdown attenuated the EODF-associated reduction in the M1/M2 macrophage ratio |
| Figure 6H | M1/M2 macrophage ratio in spinal cord tissue | SCI vs AAV-shScramble+SCI | Day 21 | 0.0789 | AAV-shScramble+SCI did not differ significantly from SCI |
| Figure 6H | M1/M2 macrophage ratio in spinal cord tissue | SCI vs EODF+SCI | Day 21 | 0.0012 | EODF significantly reduced the M1/M2 macrophage ratio |
| Figure 6H | M1/M2 macrophage ratio in spinal cord tissue | EODF+SCI vs AAV-shSrebf1+EODF+SCI | Day 21 | 0.0002 | Srebf1 knockdown attenuated the EODF-associated reduction in the M1/M2 macrophage ratio |
| Figure 7E | TUNEL-positive cells in spinal cord tissue | SCI vs AAV-shScramble+SCI | Day 3 | >0.9999 | AAV-shScramble+SCI did not differ significantly from SCI |
| Figure 7E | TUNEL-positive cells in spinal cord tissue | SCI vs EODF+SCI | Day 3 | 0.0053 | EODF significantly reduced TUNEL-positive cells |
| Figure 7E | TUNEL-positive cells in spinal cord tissue | EODF+SCI vs AAV-shSrebf1+EODF+SCI | Day 3 | 0.0050 | Srebf1 knockdown attenuated the EODF-associated reduction in TUNEL-positive cells |
| Figure 7F | TUNEL-positive cells in spinal cord tissue | SCI vs AAV-shScramble+SCI | Day 7 | 0.9419 | AAV-shScramble+SCI did not differ significantly from SCI |
| Figure 7F | TUNEL-positive cells in spinal cord tissue | SCI vs EODF+SCI | Day 7 | <0.0001 | EODF significantly reduced TUNEL-positive cells |
| Figure 7F | TUNEL-positive cells in spinal cord tissue | EODF+SCI vs AAV-shSrebf1+EODF+SCI | Day 7 | <0.0001 | Srebf1 knockdown attenuated the EODF-associated reduction in TUNEL-positive cells |
| Figure 7G | TUNEL-positive cells in spinal cord tissue | SCI vs AAV-shScramble+SCI | Day 14 | 0.4959 | AAV-shScramble+SCI did not differ significantly from SCI |
| Figure 7G | TUNEL-positive cells in spinal cord tissue | SCI vs EODF+SCI | Day 14 | 0.0059 | EODF significantly reduced TUNEL-positive cells |
| Figure 7G | TUNEL-positive cells in spinal cord tissue | EODF+SCI vs AAV-shSrebf1+EODF+SCI | Day 14 | 0.0009 | Srebf1 knockdown attenuated the EODF-associated reduction in TUNEL-positive cells |
| Figure 7H | TUNEL-positive cells in spinal cord tissue | SCI vs AAV-shScramble+SCI | Day 21 | 0.0083 | AAV-shScramble+SCI  differed significantly from SCI |
| Figure 7H | TUNEL-positive cells in spinal cord tissue | SCI vs EODF+SCI | Day 21 | <0.0001 | EODF significantly reduced TUNEL-positive cells |
| Figure 7H | TUNEL-positive cells in spinal cord tissue | EODF+SCI vs AAV-shSrebf1+EODF+SCI | Day 21 | <0.0001 | Srebf1 knockdown attenuated the EODF-associated reduction in TUNEL-positive cells |

**Supplementary Figures**

**Figure S1. Schematic representation of the animal experimental schedule.** Mice were randomly assigned to five groups: Sham, SCI, EODF+SCI, AAV-shScramble+SCI, and AAV-shSrebf1+EODF+SCI. AAV9-shScramble-GFP or AAV9-shSrebf1-GFP was administered 7 weeks before SCI induction, as indicated. After a 3-week viral-expression period, mice in the EODF+SCI and AAV-shSrebf1+EODF+SCI groups underwent 4 weeks of EODF prehabilitation, consisting of alternating 24-h fasting and 24-h ad libitum feeding. The initial 3-week period in the groups without AAV treatment represents a time-matched interval corresponding to the viral-expression period in the AAV-treated groups. Mice in the remaining groups were maintained under ad libitum feeding. The Sham group underwent laminectomy without spinal cord compression, whereas the other groups underwent SCI induction. Postoperative assessments and sample collection were performed on days 3, 7, 14, and 21 after SCI.

**Figure S2. Validation of AAV-mediated Srebf1 knockdown in the spinal cord. (A)** Representative fluorescence images of spinal cord sections showing DAPI (blue, nuclei), EGFP (green, AAV-transduced cells), and merged images 21 days after intrathecal injection of AAV9-shScramble-GFP or AAV9-shSrebf1-GFP. Scale bar = 400 μm. **(B)** Western blot analysis of SREBP1 protein expression in spinal cord tissue 21 days after AAV injection. Quantification of SREBP1 protein levels was normalized to GAPDH. Data are presented as mean ± SD (n = 3 per group). ***P* < 0.01.

**Figure S3. Body weight and food intake during EODF prehabilitation. (A)** Baseline body weight before the initiation of EODF prehabilitation in the Sham, SCI, EODF+SCI, AAV-shScramble+SCI, and AAV-shSrebf1+EODF+SCI groups. No significant intergroup difference was detected by one-way ANOVA. **(B)** Body weight changes during the 4-week EODF prehabilitation period in the indicated groups. **(C)** Daily food intake during the 4-week EODF prehabilitation period in the indicated groups. The alternating zero food intake in the EODF-treated groups reflects scheduled fasting days, whereas increased food intake on refeeding days reflects compensatory feeding. Data are presented as mean ± SD (n = 10 per group). ns, not significant.
